# Dibromido{2-[(4-bromo­phen­yl)imino­meth­yl]pyridine-κ^2^ *N*,*N*′}zinc(II)

**DOI:** 10.1107/S1600536809025653

**Published:** 2009-07-11

**Authors:** Mehdi Khalaj, Saeed Dehghanpour, Ali Mahmoudi, Shila Seyedidarzam

**Affiliations:** a Department of Chemistry, Islamic Azad University, Karaj Branch, Karaj, Iran; b Department of Chemistry, Alzahra University, PO Box 1993891176, Vanak, Tehran, Iran

## Abstract

In the title complex, [ZnBr_2_(C_12_H_9_BrN_2_)], the Zn^II^ ion is in a distorted tetra­hedral coordination environment formed by two imine N atoms of the bis-chelating *N*-heterocyclic ligand and two Br atoms. The dihedral angle between the pyridine and benzene rings is 8.04 (17)°.

## Related literature

For background information on diimine complexes, see: Small *et al.* (1998 ▶). For the use of imino­pyridine complexes as olefin polymerization catalysts, see: Ittel *et al.* (2000 ▶); Britovsek *et al.* (1999 ▶). For related structures, see Dehghanpour & Mahmoudi (2007 ▶); Dehghanpour *et al.* (2007 ▶).
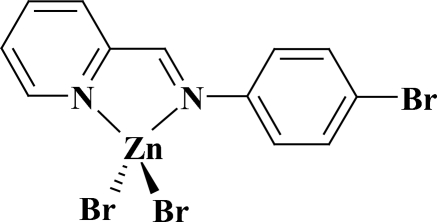


## Experimental

### 

#### Crystal data

[ZnBr_2_(C_12_H_9_BrN_2_)]
*M* *_r_* = 486.31
Triclinic, 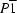

*a* = 7.7506 (13) Å
*b* = 8.7413 (16) Å
*c* = 10.9846 (18) Å
α = 89.966 (5)°
β = 72.182 (6)°
γ = 88.665 (6)°
*V* = 708.3 (2) Å^3^
*Z* = 2
Mo *K*α radiation
μ = 10.18 mm^−1^
*T* = 100 K
0.28 × 0.16 × 0.12 mm

#### Data collection

Bruker APEXII CCD area-detector diffractometer
Absorption correction: multi-scan (*APEX2*; Bruker, 2005 ▶) *T*_min_ = 0.153, *T*_max_ = 0.293
7668 measured reflections
3230 independent reflections
2703 reflections with *I* > 2σ(*I*)
*R*_int_ = 0.066

#### Refinement

*R*[*F* ^2^ > 2σ(*F* ^2^)] = 0.046
*wR*(*F* ^2^) = 0.095
*S* = 1.00
3230 reflections
163 parameters
H-atom parameters constrained
Δρ_max_ = 2.06 e Å^−3^
Δρ_min_ = −1.70 e Å^−3^

### 

Data collection: *APEX2* (Bruker, 2005 ▶); cell refinement: *APEX2*; data reduction: *APEX2*; program(s) used to solve structure: *SHELXTL* (Sheldrick, 2008 ▶); program(s) used to refine structure: *SHELXTL*; molecular graphics: *SHELXTL*; software used to prepare material for publication: *SHELXTL*.

## Supplementary Material

Crystal structure: contains datablocks global, I. DOI: 10.1107/S1600536809025653/lh2845sup1.cif

Structure factors: contains datablocks I. DOI: 10.1107/S1600536809025653/lh2845Isup2.hkl

Additional supplementary materials:  crystallographic information; 3D view; checkCIF report

## Figures and Tables

**Table:** 

Zn1—N1	2.062 (5)
Zn1—N2	2.094 (4)
Zn1—Br1	2.3310 (8)
Zn1—Br2	2.3507 (9)

**Table:** 

N1—Zn1—N2	80.62 (18)
N1—Zn1—Br1	119.57 (13)
N2—Zn1—Br1	119.19 (13)
N1—Zn1—Br2	110.14 (13)
N2—Zn1—Br2	108.91 (12)
Br1—Zn1—Br2	113.95 (3)

**Fig. 1.**
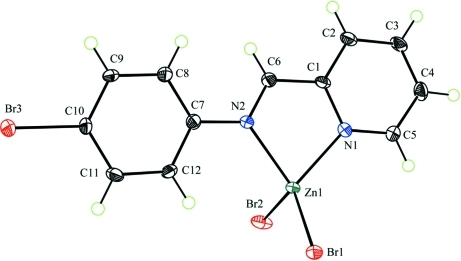
Molecular structure of (I) showing the atom-labelling scheme with thermal ellipsoids drawn at the 50% probability level.

## supplementary crystallographic information

### Comment

Complexes of iminopyridines with late transition metals have recently found a renewal of interest (Small *et al.*, 1998). The unexpected and recent discovery that such complexes in particular the imiopyridine iron(II) and cobalt (II) complexes, may act as active catalysts for olefine polymerization render them more attractive for chemists (Ittel *et al.*, 2000; Britovsek *et al.*, 1999). The title complex, (I), Fig. 1, was prepared by the reaction of ZnBr_2_ with the potentially bidentate ligand (4-bromophenyl)pyridin-2-ylmethyleneamine.

As might be expected for a four-coordinated zinc(II) complex, the metal center has a tetrahedral coordination environment. However, there are signficant distortions mainly due to the presence of the 5-membered chelate cycle: the endocyclic N1—Zn1—N2 angle [80.62 (18)°] is much narrower than the ideal tetrahedral angle of 109.5°, whereas the N1—Zn1—Br1 angle [119.57 (13)°] is much wider than the ideal tetrahedral angle. The Zn—Br and Zn—N bond dimensions compare well with the values found in other tetrahedral diimine complexes of zinc bromide (Dehghanpour & Mahmoudi, 2007; Dehghanpour *et al.*, 2007).

### Experimental

The title complex was prepared by the reaction of ZnBr_2_ and (4-bromophenyl)pyridin-2-ylmethyleneamine (molar ratio 1:1) in acetonitrile at room temperature. The solution was then concentrated under vacuum, and diffusion of diethyl ether vapor into the concentrated solution gave colourless crystals of (I) in 84% yield. Calc. for C_12_H_9_Br_3_N_2_Zn: C 29.64, H 1.87, N 5.76%; found: C 29.68, H 1.89, N 5.74%.

### Refinement

All hydrogen atoms were placed in geometrically calculated positions with C-H = 0.93Å and refined in a riding-model approximation with *U*_iso_(H) = 1.2U_eq_(C) There is a high positive residual density of 2.06 e Å^-3^ near the atom Br2 (distance 0.88%A).

### Crystal data

**Table:**

[ZnBr_2_(C_12_H_9_BrN_2_)]	*Z* = 2
*M**_r_* = 486.31	*F*(000) = 460
Triclinic, *P*1	*D*_x_ = 2.280 Mg m^−^^3^
Hall symbol: -P 1	Mo *K*α radiation, λ = 0.71073 Å
*a* = 7.7506 (13) Å	Cell parameters from 469 reflections
*b* = 8.7413 (16) Å	θ = 2.1–21.4°
*c* = 10.9846 (18) Å	µ = 10.18 mm^−^^1^
α = 89.966 (5)°	*T* = 100 K
β = 72.182 (6)°	Prism, colourless
γ = 88.665 (6)°	0.28 × 0.16 × 0.12 mm
*V* = 708.3 (2) Å^3^	

### Data collection

**Table:**

Bruker APEXII CCD area-detector diffractometer	3230 independent reflections
Radiation source: fine-focus sealed tube	2703 reflections with *I* > 2σ(*I*)
graphite	*R*_int_ = 0.066
Detector resolution: 0 pixels mm^-1^	θ_max_ = 27.5°, θ_min_ = 2.0°
ω scans	*h* = −10→10
Absorption correction: multi-scan (*APEX2*; Bruker, 2005)	*k* = −11→11
*T*_min_ = 0.153, *T*_max_ = 0.293	*l* = −14→14
7668 measured reflections	

### Refinement

**Table:**

Refinement on *F*^2^	Primary atom site location: structure-invariant direct methods
Least-squares matrix: full	Secondary atom site location: difference Fourier map
*R*[*F*^2^ > 2σ(*F*^2^)] = 0.046	Hydrogen site location: inferred from neighbouring sites
*wR*(*F*^2^) = 0.095	H-atom parameters constrained
*S* = 1.00	*w* = 1/[σ^2^(*F*_o_^2^) + (0.02*P*)^2^ + 5.8*P*] where *P* = (*F*_o_^2^ + 2*F*_c_^2^)/3
3230 reflections	(Δ/σ)_max_ < 0.001
163 parameters	Δρ_max_ = 2.06 e Å^−^^3^
0 restraints	Δρ_min_ = −1.70 e Å^−^^3^

### Special details

**Table:**

Geometry. All e.s.d.'s (except the e.s.d. in the dihedral angle between two l.s. planes) are estimated using the full covariance matrix. The cell e.s.d.'s are taken into account individually in the estimation of e.s.d.'s in distances, angles and torsion angles; correlations between e.s.d.'s in cell parameters are only used when they are defined by crystal symmetry. An approximate (isotropic) treatment of cell e.s.d.'s is used for estimating e.s.d.'s involving l.s. planes.
Refinement. Refinement of *F*^2^ against ALL reflections. The weighted *R*-factor *wR* and goodness of fit *S* are based on *F*^2^, conventional *R*-factors *R* are based on *F*, with *F* set to zero for negative *F*^2^. The threshold expression of *F*^2^ > σ(*F*^2^) is used only for calculating *R*-factors(gt) *etc*. and is not relevant to the choice of reflections for refinement. *R*-factors based on *F*^2^ are statistically about twice as large as those based on *F*, and *R*- factors based on ALL data will be even larger.

### Fractional atomic coordinates and isotropic or equivalent isotropic displacement parameters (Å^2^)

**Table:**

	*x*	*y*	*z*	*U*_iso_*/*U*_eq_	
Zn1	0.60716 (8)	0.80440 (7)	0.65671 (6)	0.01457 (15)	
Br1	0.37048 (7)	0.65625 (6)	0.63756 (5)	0.01697 (13)	
Br2	0.81619 (8)	0.67009 (7)	0.73568 (6)	0.02353 (15)	
Br3	1.15053 (7)	0.69529 (6)	−0.03171 (5)	0.01971 (14)	
N1	0.5509 (6)	1.0180 (5)	0.7411 (4)	0.0161 (9)	
N2	0.7495 (6)	0.9383 (5)	0.5020 (4)	0.0135 (9)	
C1	0.6485 (7)	1.1285 (6)	0.6650 (5)	0.0142 (10)	
C2	0.6414 (7)	1.2794 (6)	0.7039 (5)	0.0175 (11)	
H2	0.7116	1.3522	0.6505	0.021*	
C3	0.5270 (8)	1.3201 (7)	0.8248 (5)	0.0193 (11)	
H3	0.5181	1.4212	0.8530	0.023*	
C4	0.4275 (7)	1.2091 (7)	0.9018 (6)	0.0205 (12)	
H4	0.3510	1.2339	0.9830	0.025*	
C5	0.4428 (7)	1.0584 (7)	0.8567 (5)	0.0188 (11)	
H5	0.3752	0.9836	0.9093	0.023*	
C6	0.7597 (7)	1.0777 (6)	0.5374 (5)	0.0154 (10)	
H6	0.8369	1.1452	0.4824	0.018*	
C7	0.8461 (7)	0.8856 (6)	0.3766 (5)	0.0150 (10)	
C8	0.9339 (7)	0.9837 (7)	0.2784 (5)	0.0181 (11)	
H8	0.9309	1.0884	0.2941	0.022*	
C9	1.0255 (7)	0.9271 (6)	0.1580 (5)	0.0164 (11)	
H9	1.0858	0.9929	0.0931	0.020*	
C10	1.0264 (7)	0.7708 (6)	0.1348 (5)	0.0156 (11)	
C11	0.9348 (7)	0.6706 (6)	0.2296 (5)	0.0173 (11)	
H11	0.9336	0.5666	0.2125	0.021*	
C12	0.8446 (7)	0.7298 (6)	0.3514 (5)	0.0171 (11)	
H12	0.7829	0.6644	0.4160	0.020*	

### Atomic displacement parameters (Å^2^)

**Table:**

	*U*^11^	*U*^22^	*U*^33^	*U*^12^	*U*^13^	*U*^23^
Zn1	0.0158 (3)	0.0090 (3)	0.0168 (3)	−0.0017 (2)	−0.0018 (2)	−0.0003 (2)
Br1	0.0164 (3)	0.0128 (3)	0.0203 (3)	−0.00344 (19)	−0.0032 (2)	−0.0010 (2)
Br2	0.0240 (3)	0.0125 (3)	0.0378 (4)	−0.0022 (2)	−0.0148 (2)	0.0031 (2)
Br3	0.0235 (3)	0.0160 (3)	0.0157 (3)	−0.0026 (2)	0.0000 (2)	−0.0042 (2)
N1	0.016 (2)	0.013 (2)	0.018 (2)	0.0013 (17)	−0.0022 (17)	−0.0008 (18)
N2	0.014 (2)	0.010 (2)	0.015 (2)	0.0001 (16)	−0.0027 (17)	0.0004 (17)
C1	0.014 (2)	0.009 (2)	0.020 (3)	−0.0001 (19)	−0.006 (2)	0.001 (2)
C2	0.019 (3)	0.011 (3)	0.023 (3)	0.000 (2)	−0.007 (2)	−0.001 (2)
C3	0.027 (3)	0.011 (3)	0.020 (3)	0.000 (2)	−0.007 (2)	−0.006 (2)
C4	0.019 (3)	0.022 (3)	0.018 (3)	0.001 (2)	−0.002 (2)	−0.008 (2)
C5	0.017 (3)	0.017 (3)	0.019 (3)	−0.001 (2)	0.000 (2)	0.000 (2)
C6	0.014 (2)	0.014 (3)	0.017 (3)	−0.001 (2)	−0.004 (2)	0.000 (2)
C7	0.012 (2)	0.015 (3)	0.017 (3)	−0.0015 (19)	−0.0029 (19)	−0.001 (2)
C8	0.019 (3)	0.014 (3)	0.018 (3)	−0.004 (2)	0.000 (2)	−0.002 (2)
C9	0.021 (3)	0.011 (3)	0.015 (3)	−0.004 (2)	−0.002 (2)	0.002 (2)
C10	0.016 (2)	0.016 (3)	0.012 (2)	0.000 (2)	−0.0002 (19)	−0.003 (2)
C11	0.019 (3)	0.010 (3)	0.023 (3)	−0.001 (2)	−0.005 (2)	−0.001 (2)
C12	0.021 (3)	0.014 (3)	0.013 (2)	−0.005 (2)	−0.001 (2)	0.002 (2)

### Geometric parameters (Å, °)

**Table:**

Zn1—N1	2.062 (5)	C4—C5	1.397 (8)
Zn1—N2	2.094 (4)	C4—H4	0.9300
Zn1—Br1	2.3310 (8)	C5—H5	0.9300
Zn1—Br2	2.3507 (9)	C6—H6	0.9300
Br3—C10	1.898 (5)	C7—C12	1.391 (8)
N1—C5	1.333 (7)	C7—C8	1.392 (8)
N1—C1	1.361 (7)	C8—C9	1.382 (7)
N2—C6	1.291 (7)	C8—H8	0.9300
N2—C7	1.422 (7)	C9—C10	1.389 (8)
C1—C2	1.381 (7)	C9—H9	0.9300
C1—C6	1.466 (8)	C10—C11	1.389 (8)
C2—C3	1.394 (8)	C11—C12	1.399 (8)
C2—H2	0.9300	C11—H11	0.9300
C3—C4	1.374 (8)	C12—H12	0.9300
C3—H3	0.9300		
			
N1—Zn1—N2	80.62 (18)	N1—C5—C4	122.3 (5)
N1—Zn1—Br1	119.57 (13)	N1—C5—H5	118.9
N2—Zn1—Br1	119.19 (13)	C4—C5—H5	118.9
N1—Zn1—Br2	110.14 (13)	N2—C6—C1	119.3 (5)
N2—Zn1—Br2	108.91 (12)	N2—C6—H6	120.3
Br1—Zn1—Br2	113.95 (3)	C1—C6—H6	120.3
C5—N1—C1	118.2 (5)	C12—C7—C8	119.4 (5)
C5—N1—Zn1	129.7 (4)	C12—C7—N2	117.7 (5)
C1—N1—Zn1	112.0 (3)	C8—C7—N2	122.8 (5)
C6—N2—C7	121.6 (5)	C9—C8—C7	120.7 (5)
C6—N2—Zn1	111.2 (4)	C9—C8—H8	119.6
C7—N2—Zn1	126.7 (4)	C7—C8—H8	119.6
N1—C1—C2	122.5 (5)	C8—C9—C10	119.2 (5)
N1—C1—C6	115.5 (5)	C8—C9—H9	120.4
C2—C1—C6	121.9 (5)	C10—C9—H9	120.4
C1—C2—C3	118.6 (5)	C9—C10—C11	121.4 (5)
C1—C2—H2	120.7	C9—C10—Br3	118.7 (4)
C3—C2—H2	120.7	C11—C10—Br3	119.9 (4)
C4—C3—C2	119.1 (5)	C10—C11—C12	118.5 (5)
C4—C3—H3	120.4	C10—C11—H11	120.7
C2—C3—H3	120.4	C12—C11—H11	120.7
C3—C4—C5	119.2 (5)	C7—C12—C11	120.6 (5)
C3—C4—H4	120.4	C7—C12—H12	119.7
C5—C4—H4	120.4	C11—C12—H12	119.7
			
N2—Zn1—N1—C5	175.0 (5)	Zn1—N1—C5—C4	176.4 (4)
Br1—Zn1—N1—C5	56.7 (5)	C3—C4—C5—N1	0.0 (9)
Br2—Zn1—N1—C5	−78.2 (5)	C7—N2—C6—C1	176.2 (5)
N2—Zn1—N1—C1	−8.2 (4)	Zn1—N2—C6—C1	−11.5 (6)
Br1—Zn1—N1—C1	−126.5 (3)	N1—C1—C6—N2	4.8 (7)
Br2—Zn1—N1—C1	98.6 (4)	C2—C1—C6—N2	−174.1 (5)
N1—Zn1—N2—C6	10.6 (4)	C6—N2—C7—C12	172.5 (5)
Br1—Zn1—N2—C6	129.4 (3)	Zn1—N2—C7—C12	1.4 (7)
Br2—Zn1—N2—C6	−97.6 (4)	C6—N2—C7—C8	−10.4 (8)
N1—Zn1—N2—C7	−177.5 (4)	Zn1—N2—C7—C8	178.5 (4)
Br1—Zn1—N2—C7	−58.8 (4)	C12—C7—C8—C9	−2.7 (8)
Br2—Zn1—N2—C7	74.3 (4)	N2—C7—C8—C9	−179.7 (5)
C5—N1—C1—C2	0.9 (8)	C7—C8—C9—C10	1.2 (8)
Zn1—N1—C1—C2	−176.3 (4)	C8—C9—C10—C11	1.0 (8)
C5—N1—C1—C6	−177.9 (5)	C8—C9—C10—Br3	179.3 (4)
Zn1—N1—C1—C6	4.8 (6)	C9—C10—C11—C12	−1.7 (8)
N1—C1—C2—C3	−1.3 (8)	Br3—C10—C11—C12	−180.0 (4)
C6—C1—C2—C3	177.5 (5)	C8—C7—C12—C11	1.9 (8)
C1—C2—C3—C4	1.0 (8)	N2—C7—C12—C11	179.2 (5)
C2—C3—C4—C5	−0.4 (9)	C10—C11—C12—C7	0.2 (8)
C1—N1—C5—C4	−0.2 (8)		
